# Whole-Exome Sequencing and High Throughput Genotyping Identified *KCNJ11* as the Thirteenth MODY Gene

**DOI:** 10.1371/journal.pone.0037423

**Published:** 2012-06-11

**Authors:** Amélie Bonnefond, Julien Philippe, Emmanuelle Durand, Aurélie Dechaume, Marlène Huyvaert, Louise Montagne, Michel Marre, Beverley Balkau, Isabelle Fajardy, Anne Vambergue, Vincent Vatin, Jérôme Delplanque, David Le Guilcher, Franck De Graeve, Cécile Lecoeur, Olivier Sand, Martine Vaxillaire, Philippe Froguel

**Affiliations:** 1 CNRS-UMR8199, Lille Pasteur Institute, Lille, France; 2 Lille Nord de France University, Lille, France; 3 Department of Pediatrics, Saint Antoine Pediatric Hospital, Saint Vincent de Paul Hospital, Catholic University of Lille, Lille, France; 4 Department of Endocrinology, Diabetology and Nutrition, Bichat-Claude Bernard University Hospital, Assistance Publique des Hôpitaux de Paris (AP-HP), Paris, France; 5 Inserm-U695, Paris 7 University, Paris, France; 6 Inserm-U1018, Centre for research in Epidemiology and Population Health, Villejuif, France; 7 Paris-Sud 11 University, Villejuif, France; 8 EA 4489 “Perinatal Environment and Fetal Growth”, Department of Diabetology, Huriez Hospital, CHRU Lille, Lille, France; 9 Department of Genomics of Common Disease, School of Public Health, Imperial College London, Hammersmith Hospital, London, United Kingdom; Odense University Hospital, Denmark

## Abstract

**Background:**

Maturity-onset of the young (MODY) is a clinically heterogeneous form of diabetes characterized by an autosomal-dominant mode of inheritance, an onset before the age of 25 years, and a primary defect in the pancreatic beta-cell function. Approximately 30% of MODY families remain genetically unexplained (MODY-X). Here, we aimed to use whole-exome sequencing (WES) in a four-generation MODY-X family to identify a new susceptibility gene for MODY.

**Methodology:**

WES (Agilent-SureSelect capture/Illumina-GAIIx sequencing) was performed in three affected and one non-affected relatives in the MODY-X family. We then performed a high-throughput multiplex genotyping (Illumina-GoldenGate assay) of the putative causal mutations in the whole family and in 406 controls. A linkage analysis was also carried out.

**Principal Findings:**

By focusing on variants of interest (*i.e.* gains of stop codon, frameshift, non-synonymous and splice-site variants not reported in dbSNP130) present in the three affected relatives and not present in the control, we found 69 mutations. However, as WES was not uniform between samples, a total of 324 mutations had to be assessed in the whole family and in controls. Only one mutation (p.Glu227Lys in *KCNJ11*) co-segregated with diabetes in the family (with a LOD-score of 3.68). No *KCNJ11* mutation was found in 25 other MODY-X unrelated subjects.

**Conclusions/Significance:**

Beyond neonatal diabetes mellitus (NDM), *KCNJ11* is also a MODY gene (‘MODY13’), confirming the wide spectrum of diabetes related phenotypes due to mutations in NDM genes (*i.e. KCNJ11*, *ABCC8* and *INS*). Therefore, the molecular diagnosis of MODY should include *KCNJ11* as affected carriers can be ideally treated with oral sulfonylureas.

## Introduction

Maturity-onset of the young (MODY) is an early-onset non autoimmune form of diabetes with a autosomal-dominant mode of transmission [1]. MODY represents less than 2% of all non autoimmune diabetes cases and it usually develops during childhood or young adulthood [1]. This monogenic disorder is due to primary dysfunction of pancreatic beta-cells and it is rarely associated with obesity that is not required for its development, in contrast to most common forms of type 2 diabetes [1].

MODY is not a single entity as at least twelve MODY subtypes with distinct genetic aetiologies have been reported in the literature: MODY1-*HNF4A*, MODY2-*GCK*, MODY3-*HNF1A*, MODY4-*PDX1*, MODY5-*HNF1B*, MODY6-*NEUROD1*, MODY7-*KLF11*, MODY8-*CEL*, MODY9-*PAX4*, MODY10-*INS*, MODY11-*BLK* and very recently MODY12-*ABCC8* [1], [2], [3]. These distinct aetiologies are associated with substantial differences in clinical course, in terms of age of onset and level of hyperglycemia, explaining various responsiveness to treatment [3], [4]. Therefore, an early molecular diagnosis is crucial as it leads to accurate diabetes treatment and care management of the patient and his family, with estimation of diabetes risk for the asymptomatic relatives [4].

However, despite previous intensive linkage analyses and candidate gene screening, approximately 30% of MODY families from our French study cohort remain genetically unexplained (MODY-X) [1]. Recently, next-generation sequencing (NGS), in particular whole-exome sequencing (WES) which is the targeted sequencing of the human genome subset that is protein coding, has become a highly powerful and efficient strategy for identifying novel causative genes for complex disorders, although mostly monogenic so far [5]. Indeed, in less than three years, WES has been used to identify causative genes for several dozens of Mendelian disorders [5]. Recently, we provided proof-of-concept that WES can be used as a clinical tool for assessing patients presenting with an undiagnosed neonatal diabetes mellitus (NDM) that is another monogenic form of non autoimmune diabetes [6]. As WES typically identifies dozens of thousands exomic variants, a selection strategy has to be used in order to facilitate the identification of the only mutation that causes the disease [5], [6]. With regard to MODY, WES analysis of several affected and healthy relatives from a MODY family may be powerful for identifying new susceptibility genes.

In the present study, we sequenced the exome of four relatives (three MODY patients and one healthy member) from a large French MODY-X family. After a filtering strategy of the identified variants and an additional genotyping of putatively causal mutations in the extended MODY-X family, we identified the thirteenth MODY gene.

## Results

**Figure 1:**
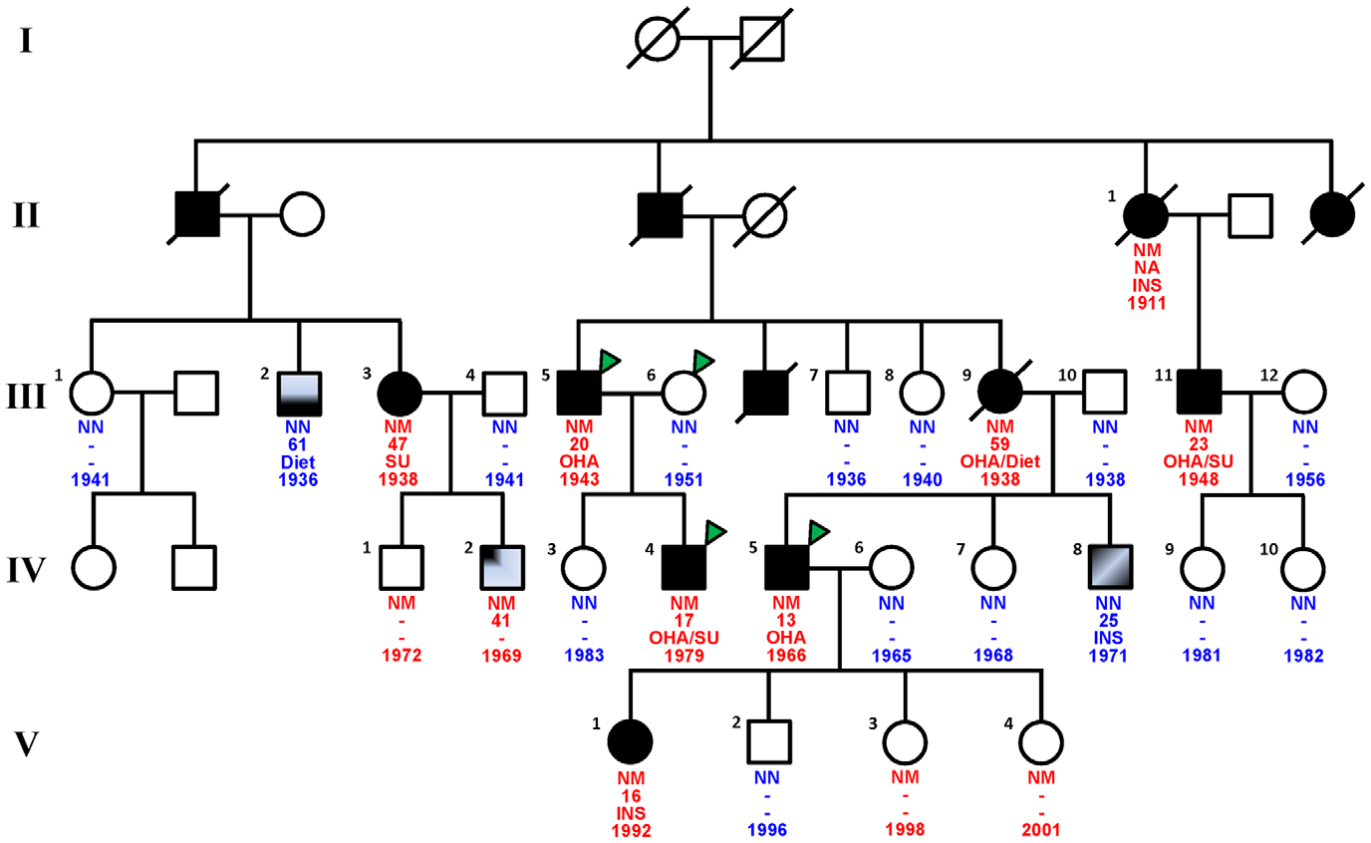
Pedigree of the family showing diabetes status of each member, as well as genetic status, age of diagnosis, treatment and date of birth. With regard to the genetic status, NM denotes the presence of the heterozygous *KCNJ11* p.Glu227Lys mutation and NN denotes the absence of mutation at the same locus. Circles represent female participants and squares male participants. A slash through the symbol indicates that the family member is deceased. Black symbols indicate patients with non autoimmune diabetes. The half-filled and quarter-filled symbols indicate individuals with impaired glucose tolerance and impaired fasting glucose, respectively. The black symbols with a white diagonal denote patients with type 1 diabetes. Green arrows point to members for whom the whole-exome was sequenced. ***INS***, insulin; ***OHA***, oral hypoglycaemic agents; ***SU***, sulfonylurea.

We analysed a four-generation MODY-X family including 12 affected members, one member with impaired fasting glucose, one member with impaired glucose tolerance, one member with documented type 1 diabetes and 22 non-affected relatives (**Figure 1**). Of note, no member of the family showed NDM.

We sequenced the exome of four members from this family: a diabetic patient diagnosed at 17 years, his diabetic father diagnosed at 20 years, his non-affected mother and his diabetic cousin diagnosed at 13 years (**Figure 1**, **Table 1**).

**Table 1:** Clinical and molecular genetic characteristics of all studied members from the French MODY family.

Member #	Mutation carrier	Date of birth	Diabetes status	BMI [kg/m^2^]	Age at diagnosis [years]	Current treatment^a^	FPG (age) [mmol/l]	PG 2 h after OGTT(age) [mmol/l]	HbA1c (age) [%]
**II1**	Yes	1911	T2D	NA	NA	INS	13.9 (87)	–	NA
**III1**	No	1941	NG	25.6	–	–	4.9 (56)	6.5 (56)	NA
**III2**	No	1936	IGT	25.0	61	Diet	6.0 (61)	8.1 (61)	NA
**III3**	Yes	1938	T2D	21.5	47	SU	5.0 (73)	–	5.7 (73)
**III4**	No	1941	NG	23.5	–	–	4.5 (55)	4.4 (55)	NA
**III5**	Yes	1943	T2D	29.0	20	OHA	8.1 (67)	–	6.2 (67)
**III6**	No	1951	NG	NA	–	–	5.4 (46)	4.5 (46)	NA
**III7**	No	1936	NG	28.4	–	–	5.7 (62)	5.4 (62)	NA
**III8**	No	1940	NG	23.6	–	–	5.4 (57)	4.9 (57)	NA
**III9**	Yes	1938	T2D	23.7	59	OHA/Diet	7.5 (59)	–	NA
**III10**	No	1938	NG	23.9	–	–	5.4 (65)	NA	NA
**III11**	Yes	1948	T2D	26.5	23	OHA/SU	9.4 (63)	–	8.6 (63)
**III12**	No	1956	NG	24.4	–	–	5.1 (40)	4.8 (40)	NA
**IV1**	Yes	1972	NG	27.7	–	–	5.3 (39)	4.4 (39)	NA
**IV2**	Yes	1969	IFG	NA	41	–	6.3 (41)	7.3 (41)	NA
**IV3**	No	1983	NG	NA	–	–	4.7 (27)	NA	5.0 (27)
**IV4**	Yes	1979	T2D	21.7	17	OHA/SU	7.5 (32)	–	6.8 (32)
**IV5**	Yes	1966	T2D	19.9	13	OHA	9.3 (45)	–	7.1 (45)
**IV6**	No	1965	NG	19.3	–	–	5.0 (45)	NA	NA
**IV7**	No	1968	NG	20.1	–	–	4.7 (42)	3.8 (42)	NA
**IV8**	No	1971	T1D	NA	25	INS	16.2 (26)	–	NA
**IV9**	No	1981	NG	17.9	–	–	4.5 (15)	5.5 (15)	NA
**IV10**	No	1982	NG	18.2	–	–	4.7 (14)	NA	NA
**V1**	Yes	1992	T2D	18.4	16	INS	7.2 (19)	–	NA
**V2**	No	1996	NG	17.2	–	–	5.0 (14)	NA	NA
**V3**	Yes	1998	NG	17.1	–	–	5.1 (12)	NA	NA
**V4**	Yes	2001	NG	13.2	–	–	5.0 (10)	NA	NA

a or last treatment for deceased people;

***BMI***, body mass index; ***FPG***, fasting plasma glucose; ***PG***, plasma glucose; ***OGTT***, oral glucose tolerance test; ***HbA1c***, glycated hemoglobin; ***NA***, not available; ***NG***, normoglycaemic; ***IFG***, impaired fasting glucose; ***IGT***, impaired glucose tolerance; ***T2D***, type 2 diabetes (non autoimmune diabetes); ***T1D***, type 1 diabetes (autoimmune diabetes); ***INS***, insulin; ***OHA***, oral hypoglycaemic agents; ***SU***, sulfonylurea.

Of note, no member of the family showed NDM.

After target enrichment, whole exome DNA libraries from the four relatives were sequenced in 76 bp paired-end reads, using two channels of the GAIIx, achieving a mean depth of coverage between 90.8 and 125.7× (**Table 2**). Depending on the Agilent capture we used (38 Mb or 50 Mb) and probably on the DNA quality, we found between 45,124 and 92,768 variants per exome (**Table 2**). By focusing on variants of interest, *i.e.* non-synonymous and splice-site variants, gains of stop codon or frameshift mutations, it remained between 7,925 and 11,632 variants, including 540 and 882 variants not reported in the database dbSNP130, respectively (**Table 2**). Subsequently, we identified 839 variants of interest present in the three affected relatives (IV4, III5 and IV5, **Figure 1**) and not present in the non-affected family member (III6, **Figure 1**), of which 69 were not reported in the database dbSNP130 (**Table 3**). Therefore, it was probable that the causal mutation for MODY was included in this set of 69 mutations. However, we found that the depth of coverage was not uniform, depending on the DNA sample (and not only on the Agilent capture version). Indeed, for instance, we identified a total of 210 variants of interest (of which 34 were not reported in dbSNP130) in the affected member III5, which could not be called in the affected member IV4, as depth of coverage was below 8× at the related loci (see combinations #4 and #6 in **Table 3**). Therefore, at this stage, we were not able to know if the affected member IV4 also carried this set of mutations. As the exome of the affected member IV5 was performed with the Agilent capture ‘50 Mb’ (instead of ‘38 Mb’ for the affected members IV4 and III5), we identified lots of variants for this family member (2,625 variants of interest of which 209 were not reported in dbSNP130) that could not be called in the affected members IV4 and III5 (see combination #7 in **Table 3**). Therefore, at this stage, it was also impossible to know if the two other affected members carried these mutations. Finally, by taking into account all the possible combinations in the three affected members, we identified a total of 324 putatively causal mutations for MODY (not present in the non-affected member III6 and not reported in dbSNP130) (**Table 3**).

**Table 2:** Number of variants identified through the WES analysis of the four DNA samples.

Members		IV4 (affected)	III5 (affected)	III6 (control)	IV5 (affected)
Agilent capture used (Mb)		38	38	38	50
Sequenced regions with coverage ≥8× (Mb)		34.2	34.2	35.2	45.0
Mean depth of coverage (×)		90.8	104.4	125.7	95.9
Total targeted variants^a^	Homozygous (**Novel**)	18,163 (**531**)	20,461 (**817**)	26,475 (**1,301**)	36,637 (**1,999**)
	Heterozygous (**Novel**)	26,961 (**3,048**)	30,574 (**4,195**)	39,056 (**5,769**)	56,131 (**7,078**)
Splice-site variants (including indels)	Homozygous (**Novel**)	526 (**9**)	577 (**9**)	623 (**12**)	738 (**12**)
	Heterozygous (**Novel**)	691 (**57**)	777 (**77**)	951 (**96**)	1,099 (**109**)
Non-synonymous variants	Homozygous (**Novel**)	2,702 (**24**)	2,952 (**26**)	3,119 (**34**)	3,735 (**36**)
	Heterozygous (**Novel**)	3,954 (**441**)	4,417 (**504**)	4,845 (**528**)	5,917 (**666**)
Non-synonymous variants leadingto a gain of STOP codon	Homozygous (**Novel**)	8 (**1**)	9 (**0**)	10 (**2**)	16 (**1**)
	Heterozygous (**Novel**)	40 (**4**)	43 (**9**)	53 (**7**)	82 (**14**)
Frameshift variants	Homozygous (**Novel**)	3 (**3**)	4 (**4**)	13 (**13**)	37 (**36**)
	Heterozygous (**Novel**)	1 (**1**)	2(**2**)	3 (**2**)	8 (**8**)
Frameshift variants leadingto a gain of STOP codon	Homozygous (**Novel**)	0 (**0**)	0 (**0**)	0 (**0**)	0 (**0**)
	Heterozygous (**Novel**)	0 (**0**)	1 (**1**)	0 (**0**)	0 (**0**)

a This includes all identified variants (including insertion or deletion) that reach the quality threshold and with depth of coverage ≥8×; ***Novel*** means not present in the public database dbSNP130; ***Indel***, insertion or deletion.

**Table 3:** Estimation of number of variants to be assessed by genotyping in the extended family and in controls.

	Depth of coverage				
Combination	Member IV4	Member III5	Member IV5	Number of variants of interest^a^, not present in member III6 = (1)	(1) - variants present in dbSNP130
**1**	≥8×	≥8×	≥8×	839	69
**2**	≥8×	≥8×	<8×	13	7
**3**	≥8×	<8×	≥8×	16	2
**4**	<8×	≥8×	≥8×	121	9
**5**	≥8×	<8×	<8×	3	3
**6**	<8×	≥8×	<8×	89	25
**7**	<8×	<8×	≥8×	2,625	209
			**TOTAL:**	**3,706**	**324**

a Variants of interest are non-synonymous mutations, splice site mutations, gains of STOP codon. No frameshift mutation was found in any of the combinations.

By using an Illumina GoldenGate assay, we assessed the presence of this set of 324 mutations in the whole family (23 additional DNA samples were available, **Figure 1**) and in 406 European adults (>47 years old), from the French D.E.S.I.R. study, which presented with normal fasting plasma glucose. Among mutations that were not present in the 406 controls, only one mutation (at a heterozygous state) was present in the eight relatives with overt non autoimmune diabetes (II1, III3, III5, III9, III11, IV4, IV5 and V1, **Table 1**, **Figure 1**). This mutation was also carried by a prediabetic member (IV2), his non-diabetic brother (IV1) and two non-diabetic children (V3 and V4) (**Table 1**, **Figure 1**). All the other non-diabetic members (III1, III2, III4, III6, III7, III8, III10, III12, IV3, IV6, IV7, IV9, IV10 and V2) and the type 1 diabetic member (IV8) did not carry the mutation (**Table 1**, **Figure 1**). This mutation is a non-synonymous variant (c.679G>A; p.Glu227Lys) located in the *KCNJ11* gene (NM_000525.3).

**Figure 2:**
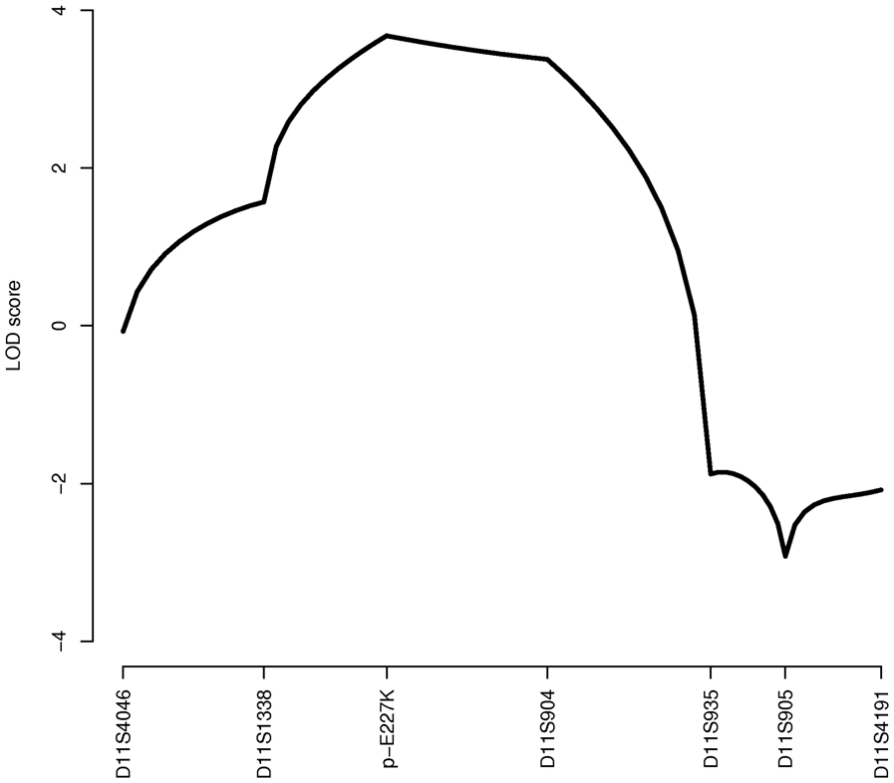
Multipoint linkage analysis following a dominant parametric model in the French pedigree. The positions of the genetic markers are ordered from chromosome 11p15.5 to 11q12.2 (chr11∶1,820,211–59,856,421; positions given according to human genome assembly GRCh37/hg19).

We then performed a linkage analysis using a dominant parametric model based on three age-dependent liability classes (<13, 13–39 and ≥40 years old). We found a maximum LOD score of 3.68 at the locus of *KCNJ11* p.Glu227Lys mutation (chromosome 11p15.1), following a two-point or multipoint analysis (**Figure 2**). Under a non-parametric linkage (NPL) model, we found a maximum NPL score of 4.65 (*P*  = 0.001) and 4.42 (*P*  = 0.00005) at the locus of the mutation, following two-point and multipoint analyses, respectively. Therefore, it is highly probable that *KCNJ11* p.Glu227Lys mutation is causal for MODY in the analyzed pedigree. Of note, we previously missed this linkage peak [7] as family member IV8 had been erroneously considered as a MODY patient while he had type 1 diabetes. Indeed, in mid 2011, we found a positivity for GAD autoantibodies from a serum sample collected in 1996, and the patient was found to carry HLA class II (DRB1*04) alleles which confer susceptibility to type 1 diabetes.

We sequenced *KCNJ11* in 22 other French MODY-X unrelated probands, and we did not identify any non-synonymous variant that was not reported in the public database dbSNP130 (data not shown), implying that *KCNJ11* is rarely mutated in French MODY families.

## Discussion

The present study based on WES, high-throughput multiplex genotyping and linkage analysis, unambiguously identified the thirteenth MODY gene, *KCNJ11* that encodes pore-forming KIR6.2 subunit of the pancreatic beta-cell ATP-dependent potassium (K-ATP) channel. The K-ATP channel that is a hetero-octamer consisting of four KIR6.2 subunits and four sulfonylurea receptor 1 (SUR1) subunits (encoded by *ABCC8*), links cellular nutrient metabolism to membrane electrical activity, which reflects the energy status of the beta-cell, and thereby plays a key role in insulin secretion [8]. Approximately 35% of patients with NDM have a gain-of-function mutation in *KCNJ11* or in *ABCC8* [1]. Importantly, most NDM patients who carry a K-ATP channel mutation can successfully be transferred from insulin therapy to oral sulfonylureas [9], [10]. Therefore, in this newly elucidated French MODY family, it will be crucial to adjust the treatment of all relatives with non autoimmune diabetes, in particular member V1 who is currently treated by insulin (**Table 1**, **Figure 1**) and members III11 and IV5 who show a quite bad metabolic control (**Table 1**, **Figure 1**). Importantly, the affected member III3 who also carries the mutation and who has always been treated by oral sulfonylureas, shows a perfect metabolic control at 73 years (HbA1c  = 5.7% and fasting plasma glucose  = 5.0 mmol/l, after a duration of diabetes of diabetes; **Table 1**, **Figure 1**). It confirms recent data obtained in NDM patients showing that after a follow up of 68 months, sulfonylureas are still active in patients with *KCNJ11* mutations, ruling out the hypothesis of a long term toxicity of this drug on pancreatic beta-cells [11].

The presently identified *KCNJ11* p.Glu227Lys mutation has already been reported in the literature [12], [13], [14], [15], [16], [17], [18]. The mutation is known to cause transient NDM (with diabetes relapse before 10 years old in most cases) and it has been reported to arise *de novo* [13], [14] or to be transmitted from one of both parents who presented with a transient NDM [13] or with other form of non autoimmune young-onset diabetes [12], [15], [17], [18]. A study reported that the p.Glu227Lys mutation has a functional effect: it reduces the sensitivity of the K-ATP channel to inhibition by MgATP, and enhances the intrinsic open probability of the K-ATP channel, in *Xenopus* oocytes expressing the mutant (at the heterozygous state) [16].

*KCNJ11* gene screening is currently indicated by guidelines in all patients who present with diabetes diagnosed before 6–12 months of age [6], [19], [20]. Some studies reported that families of patients with a transient or permanent form of NDM due to a *KCNJ11* mutation can also include other carriers in the family with childhood or later-onset diabetes (age of diagnosis before 30 years) [17], [21], [22]. However, no previous study has ever described a family with a well-defined MODY due to a *KCNJ11* mutation. Furthermore, our MODY family does not include any member who presented with NDM. Therefore, since the affected carriers of a *KCNJ11* mutation should ideally receive oral sulfonylureas, the routine genetic diagnosis of MODY families should now include *KCNJ11*, even if the prevalence of *KCNJ11* mutations in MODY seems quite low. Indeed, we did not identify any other *KCNJ11* mutation in our MODY-X families. Furthermore, Bowman *et al.* sequenced both *ABCC8* and *KCNJ11* genes in 85 patients who had been referred for genetic testing for MODY and who were sensitive to (or treated with) oral sulfonylureas, and they did not identify any mutation in *KCNJ11* [2]. Even if we actually found one MODY family only with a *KCNJ11* mutation, so far, the true number might be larger, especially in atypical MODY families (with young adult onset patients). Assessing a putative MODY13 in such families can be very helpful for a more personalized treatment of their specific form of diabetes.

Importantly, our study firmly confirms that *KCNJ11* mutations can be associated with a large spectrum of diabetes phenotypes and can be not totally penetrant. Indeed, member IV1 of our French MODY family, who carries the *KCNJ11* p.Glu227Lys mutation, has normal fasting plasma glucose level at 39 years (5.3 mmol/l, **Table 1**, **Figure 1**). This phenotypic spectrum of diabetes has also been reported in carriers of a mutation in *ABCC8* [2], [23] or in the insulin (*INS*) gene [24], [25], [26], [27], which both represent, with *KCNJ11*, the most frequently mutated genes in patients with NDM [1]. Epigenetic effects or other modifier genetic effects could explain the substantial difference in both diabetes onset and clinical expression between NDM and MODY patients.

Some lessons can be obtained from the present study. At present, although the WES technology is highly powerful and quickly led to the elucidation of several dozens of syndromes, it does not provide a perfect sequencing. Indeed, even with a mean depth of coverage higher than 90×, WES is not uniform between samples and has lots of gaps. Therefore, when the analysis is based on WES from several affected relatives, an additional genotyping is necessary in order to avoid any false negative result. For a molecular diagnosis of MODY based on NGS, a targeted sequencing (by hybridization or using a droplet-based PCR technology) of all MODY genes, including NDM genes as they are also responsible for other forms of diabetes, may be more accurate at present. As WES technology is improving day after day, we can expect this technique to be much easier in the near future.

## Materials and Methods

### Study Participants

The French MODY-X family (named F725) was recruited by the CNRS UMR8199 unit in 1996 [28], and it has regularly been extended since this date, with updated clinical data for all relatives (last update was performed in mid 2011) (**Table 1**). A pedigree of the family is shown in **Figure 1**. Of note, no member of the family showed NDM. In the proband of the family, we previously searched for mutations in the known susceptibility genes for MODY: *HNF4A*, *GCK*, *HNF1A*, *HNF1B*, *PDX1*, *KLF11*, *BLK*, *INS* and *ABCC8.* All these genetic screenings were performed by a standard Sanger sequencing method. No putatively causal mutations were identified.

We assessed the candidate mutations in 406 normal glucose French adults (>47 years old) from the Data from the Epidemiological Study on the Insulin Resistance Syndrome (D.E.S.I.R.) cohort. The D.E.S.I.R. study is a longitudinal French general population cohort, fully described elsewhere [29].

For *KCNJ11* gene screening, we used DNA samples from a total of 22 unrelated French probands presenting with MODY-X following these criteria: i/presence of overt diabetes in at least three consecutive generations with a dominant transmission and/or at least two diabetic patients diagnosed before age 25 years; ii/no requirement for insulin therapy during the first two years after diagnosis, or measurable C-peptide several years after diagnosis; and iii/absence of auto-immunity markers. These subjects were recruited by the CNRS UMR8199 unit. Glycemic status for non-MODY individuals was defined according to 1997 American Diabetes Association or 1999 World Health Organization criteria: normal glucose defined as fasting glucose <6.1 mmol/l without hypoglycemic treatment; impaired fasting glucose defined as fasting glucose between 6.1 and 7.0 mmol/l, without hypoglycemic treatment; impaired glucose tolerance defined as glucose concentration 2 h after an oral glucose load between 7.8 and 11.1 mmol/l; and T2D defined as fasting glucose ≥7.0 mmol/l and/or treatment with antidiabetic agents.

All DNA samples used for the present study were extracted from blood.

The study was approved by the local ethics committees (CNIL [Commission Nationale de l’Informatique et des Libertés] #901060 and CCPPRB [Comité Consultatif de Protection des Personnes dans la Recherche Biomédicale] of Lille and Paris). Each participant gave written informed consent. With regard to child participant, both parents gave written informed consent for the genetic testing of their child.

### Targeted Capture and Massive Parallel Sequencing

Approximately 99.18% of CCDS exons or 98.75% of RefSeq exons from 3 µg of genomic DNA were captured using the Agilent SureSelect Human All Exon Kit (‘38 Mb’ kit for members III5, III6 and IV4, and ‘50 Mb’ kit for member IV5), following the manufacturer’s protocols (Agilent, Santa Clara, CA, USA). Briefly, DNA was sheared by acoustic fragmentation (Bioruptor NGS, Diagenode, Liège, Belgium) and purified using Agencourt AMPure XP beads (Beckman Coulter, Fullerton, CA, USA). The quality of the fragmentation and purification was assessed with the Agilent 2100 Bioanalyzer. The fragment ends were repaired and adaptors were ligated to the fragments. The resulting DNA library was purified using the Agencourt AMPure XP beads, amplified by PCR and captured by hybridization to the biotinylated RNA library baits. Bound genomic DNA was purified with streptavidin coated magnetic Dynabeads (Invitrogen, Carlsbad, CA, USA) and re-amplified. The whole-exome DNA library was sequenced on the Illumina Genome Analyzer IIx (GAIIx) in 76 base-pairs (bp) paired-end reads and using two channels (Illumina, San Diego, CA, USA).

### Sequence Reads Mapping and Variant Calling

Sequence reads were mapped to the reference human genome (UCSC NCBI36/hg18) using the ELANDv2 software (Illumina). Variant detection was performed with the CASAVA software (version 1.6, Illumina) and filtered to fit a CASAVA quality threshold ≥10 and depth of ≥8×. CASAVA filters duplicate reads and reads without matched pairs.

### Genotyping of the 324 Candidate Mutations in the Whole Family and in Controls

To validate mutations obtained by WES, we used a multiplex high-throughput single nucleotide polymorphism (SNP) detection assay based on Golden Gate technology (Illumina), allowing the genotyping of 324 SNPs in a total of 433 DNA samples (27 members of the MODY family and 406 normal glucose individuals from the D.E.S.I.R. cohort study). The two-day protocol was performed according to the manufacturer’s recommendations. Veracode BeadXpress Reader scanned each 96-well reaction plate (Illumina). All the generated data were processed using GenomeStudio 1.8 Software (Illumina) to infer all SNP genotypes via three clusters on a graph based on the fluorescence obtained.

### Sanger Sequencing

Approximately 8% of the mutations found in WES could not be designed by the Illumina BeadXpress Assay Design Tool because of technical issues (e.g. another SNP very closed to the targeted SNP, high GC content, CNV or repeat region). In these cases, confirmation of SNPs was realized by Sanger sequencing on a 3730×l DNA Analyser (Applied Biosystems, Foster City, CA, USA). Sanger sequencing was also used to double-check p.Glu227Lys mutation in *KCNJ11*. A standard protocol was followed and primer designs and PCR conditions can be provided upon request. Sequencing reads were assembled and analyzed with Variant Reporter software (Applied Biosystems).

### Linkage Analysis

Two-point and multipoint linkage analyses were performed in the extended family using both parametric and non-parametric methods.

For the parametric model, initial genetic data obtained for the pedigree from a previous genome-wide scan study [7], as well as the *KCNJ11* p.Glu227Lys genotypes, were analyzed using the MLINK and SIMWALK2 programs, based on several age-dependent liability classes (LINKAGE package), as previously described [30]. The model used a disease allele frequency of 0.002 and three age-dependent liability classes: <13, 13–39, and ≥40 years, with age-dependent penetrances for one or two copies of the disease allele of 0.10, 0.60 and 0.90, respectively. The penetrances for the homozygous non-susceptible genotypes were assumed to be 0.00, 0.03 and 0.03 for the three liability classes, respectively.

We used the program Allegro.2.0 f [31] to compute a non-parametric linkage (NPL) score, which allowed us to assess linkage without specifying a mode of inheritance in the extended family. This statistic is based on the transmission of an allele according to the genotypes in the family relatives. Thus, a vector of inheritance is defined, which is used to determine the number of alleles shared between pairs of affected relatives in the family. Allele frequencies were estimated with an estimation-maximisation algorithm. The genetic maps used in the multipoint analyses were built according to the Genethon data (with the genetic distances estimated in cM Haldane map).
